# Reimbursement measures in European countries – findings of a bibliometric literature review

**DOI:** 10.1186/2052-3211-8-S1-P21

**Published:** 2015-10-05

**Authors:** Sabine Vogler, Nina Zimmermann, Antonio Olry de Labry, Jaime Espin

**Affiliations:** 1 WHO Collaborating Centre for Pharmaceutical Pricing and Reimbursement Policies, Health Economics Department, Gesundheit Österreich GmbH (Austrian Public Health Institute), Vienna, 1010, Austria; 2 Escuela Andaluza de Salud Pública / Andalusian School of Public Health, Campus Universitario de Cartuja, Granada, 18011, Spain

## Background

Policy-makers aim to achieve the partially conflicting objectives of equitable access to medicines, cost containment and sustainable funding as well as reward for innovation. To do so, a range of policy options is available that has been extended in recent years to meet new challenges.

## Objectives

To identify existing pharmaceutical reimbursement policy options in European countries

## Methods

A literature review was carried out using thesaurus and free terms in several databases and grey literature. Setting: Out-patient and in-patient sectors including possible measures at the interface of out-patient and in-patient sectors and stakeholders involved: State (as regulator), third party payers and patients (funders); pharmaceutical industry. Inclusion criteria: Studies or documents published between 1993 and February 2013 in all European Union (EU) languages performed in all 28 EU Member States and European Economic Area.

## Results

In total 244 publications were selected, 61% of the selected studies were published between 2007 and 2011. Most literature referred to a sole country, particularly to large countries such as the UK and Germany. Descriptive work constituted a major body of literature; an impact assessment of policy measures was undertaken in 29% of the publications.

The five reimbursement policies most frequently mentioned were: co-payments (mentioned in 51% of the selected publications); reimbursement rates (45%); reference price systems (43%); positive lists (43%) and the reimbursement process (40%). More than every second publication addressed either HTA or pharmaco-economics. Generic substitution, reimbursement reviews, tendering and INN prescribing were identified in 35%–22% of the included publications. Nine per cent of the publications referred to managed-entry agreements, and 7% mentioned value-based pricing. Reimbursement policies addressed in low frequency were auction-like systems, profit control or delisting from reimbursement.

## Conclusions

Standard elements of reimbursement systems in European countries were identified in several publications, whereas newer policy options were covered less frequently in literature. Discussions about tools on how to best assess the value of (new) medicines are recurring in literature.

The literature reflects, with some publication delay, the approaches of policy-makers with regard to pharmaceutical reimbursement measures. In the 1990s and early years of the new millennium, descriptive studies about national reimbursement systems were predominant, supplemented, at a later stage, by descriptions and analyses of generic policies. In the new millennium, discussions about value assessments and the importance of HTA and pharmaco-economics, frequently understood as a contrast to the pricing policy of external price referencing, were found. Policy options for new, high-priced medicines were particularly addressed in literature of recent years.

